# Static-Aligned Piezoelectric Poly (Vinylidene Fluoride) Electrospun Nanofibers/MWCNT Composite Membrane: Facile Method

**DOI:** 10.3390/polym10090965

**Published:** 2018-09-01

**Authors:** Nader Shehata, Eman Elnabawy, Mohamed Abdelkader, Ahmed H. Hassanin, Mohamed Salah, Remya Nair, Sameer Ahmad Bhat

**Affiliations:** 1 Department of Engineering Mathematics and Physics, Faculty of Engineering, Alexandria University, Alexandria 21544, Egypt; m.salah@mena.vt.edu; 2 Center of Smart Nanotechnology and Photonics (CSNP), SmartCI Research Center, Alexandria University, Alexandria 21544, Egypt; ch.eman.elnabawy@gmail.com (E.E.); mohamed.fawzy@mena.vt.edu (M.A.); ahassanin2003@yahoo.com (A.H.H.); 3 The Bradley Department of Electrical and Computer Engineering, Virginia Tech, Blacksburg, VA 24061, USA; 4 Kuwait College of Science and Technology (KCST), Doha District 13133, Kuwait; r.nair@kcst.edu.kw (R.N.); s.bhat@kcst.edu.kw (S.A.B.); 5 Department of Physics, Faculty of Science, Fayoum University, Fayoum 63514, Egypt; 6 Department of Electrical Engineering, Faculty of Engineering, Alexandria University, Alexandria 21544, Egypt; 7 Department of Textile Engineering, Faculty of Engineering, Alexandria University, Alexandria 21544, Egypt

**Keywords:** piezoelectric, PVDF, carbon nanotubes, electrospinning, nanofibers

## Abstract

Polyvinylidene Fluoride (PVDF) piezoelectric electrospun nanofibers have been intensively used for sensing and actuation applications in the last decade. However, in most cases, random PVDF piezoelectric nanofiber mats have moderate piezoelectric response compared to aligned PVDF nanofibers. In this work, we demonstrate the effect of alignment conducted by a collector setup composed of two-metal bars with gab inside where the aligned fiber can be formed. That is what we called static aligned nanofibers, which is distinct from the dynamic traditional technique using a high speed rotating drum. The two-bar system shows a superior alignment degree for the PVDF nanofibers. Also, the effect of added carbon nanotubes (CNTs) of different concentrations to PVDF nanofibers is studied to observe the enhancement of piezoelectric response of PVDF nanofibers. Improvement of β-phase content of aligned (PVDF) nanofibers, as compared to randomly orientated fibers, is achieved. Significant change in the piezoelectricity of PVDF fiber is produced with added CNTs with saturation response in the case of 0.3 wt % doping of CNTs, and piezoelectric sensitivity of 73.8 mV/g with applied masses down to 100 g.

## 1. Introduction

Piezoelectric polymer is a highly flexible type of polymer which can be deformed by stretching, compression, or bending, thereby producing an electrical polarization in response to applied stress. The polarization is caused by a reorientation of the net dipole moment of polymer polar groups into a particular direction [1,2,3,4,5]. Several types of semicrystalline polymers such as PVDF, Polyamides [6], Polyureas, and liquid crystal have been used for piezoelectric applications due to their high flexibility, strength, and impact resistance. Among different piezoelectric polymers, PVDF and its copolymer with tetraflouoroethylene (TFE) and trifluoroethylene (TrFE) represent the state of the art of piezoelectric fluropolymers in the last decade. The arrangement of the PVDF repeat unit (-CH_2_-CF_2_-) can affect the polymer properties, depending on the chain conformation either α, β or γ phases [7]. If the PVDF crystallization is accompanied with parallel dipole formation, an increase in the β phase content could result, and subsequently, a piezoelectric response is produced [8,9,10,11].

Electrospinning is one of the most popular techniques for nanofiber (NF) fabrication [12,13,14,15], in which an increasing of β phase content can originate through the stretching of polymers in a high electric field [16,17]

The impact of the addition of carbon nanotubes (CNTs) on the crystal structure and piezoelectric properties of PVDF nanofiber has been widely studied. PVDF/CNTs nanofiber composite presents high β phase formation, high electrical signal, and improved mechanical properties that make CNTs nanofiber composite a good candidate for a wide range of sensing applications. The increase of linear behavior in output voltages of piezoelectric signals for aligned and randomly-oriented electrospun PVDF/CNTs membranes was investigated with applied loading up to 350 N, and showed high stability during cyclic dynamic compression and tension [18].

Several techniques have been investigated to increase the degree of alignment of polymeric nanofiber through electrospinning [19]; continuous electrospinning of aligned fiber through a copper wire drum collector was studied, as the alignment of nanofiber was formed by electrostatic interaction which allowed the fiber to be stretched across the gap between the copper wires [20]. A magnetic field was used to align fluorescent nanowires of nickel coated with porphyrin in fluid solutions. Applying an external magnetic field through the collector region of the electrospinning setup was intensively studied for fabrication of aligned nanofibers with large areas and high membrane thickness. Charged fibers can be spun onto the collector and stretched across a gap of two opposite magnetic poles along the directions of the magnets in order that well-ordered fibers may easily be formed [21,22]. Axially-aligned organic polymers, ceramics, and polymer ceramic composite were obtained using two conductive silicon rods separated by a gab, inside which aligned fiber can be spun with a width ranging from micrometers to a few centimeters [23].

Electrospun piezoelectric polymers have the potential to be used in various applications [24,25,26,27]. Piezoelectric nanofibers of BNT-ST (0.78 Bi, 0.5 Na, 0.5 TiO_3_-0.22 SrTiO_3_)/PVDF-TrFE composites were fabricated by the electrospinning method, observing the effect of the drum collector rotation speed from 0 to 1500 rpm on the degree of alignment of electrospun nanofiber. An increase of nanofiber orientation resulted that increased the piezoelectric output [28]. Air pressure sensors based on PVDF nanofibers were fabricated using a double-plate collector. The enhancement of piezoelectric performance was investigated through polyethylene terephthalate (PET) top-plate collector, consisting of two pieces of the same sheet of PET with a certain gap, and bottom-plate, which was a whole piece of PDMS sheet. The orientation of PVDF fiber became more aligned by increasing the thickness of the PET sheet, thereby producing a higher piezoelectric signal [29]. Simulation of a human finger was investigated through a fingertip sensor designed from a metal bar as the bone, a body, and a skin layer of silicon rubber. PVDF-based sensors were embedded in the body of fingertip, and the skin layer was used as a receptor that could distinguish between five different materials by collecting texture information through pushing the objects [30].

In this study, Piezoelectric PVDF/CNTs nanofibers were fabricated through electrospinning. Aligned fiber through 2-metal bars was investigated by studying the effect of the alignment and CNT concentration on the piezoelectric response. This alignment technique is static and alternates the traditional rotating drum alignment procedure. This is important for further study of scaling-up the generated nanofibers mat. The fabricated mats of the collected aligned and random nanofibers were analyzed by scanning electron microscope (SEM), and the β phase content was calculated by Fourier transform (FT-IR) analysis. The crystal phase of the piezoelectric nanofibers was investigated by X-ray diffraction (XRD) analysis [31]; COMSOL finite-element software was used for determining the electric field orientation. The piezoelectric response of the generated nanofibers mats was detected through a simple setup, which applied weights to a sandwich of PVDF nanofiber mats covered by two foil thin sheets. Then, the generated voltage was detected through a high impedance oscilloscope.

## 2. Materials and Methods

### 2.1. Materials

The main precursor of Polyvinylidene Fluoride (PVDF) (Kynar®, King of Prussia, PA, USA) is supplied by ARKEMA. Multi-walled carbon nanotubes (CNTs), from Cheaptubes Inc. (Cambridgeport, VT, USA) are added within different weight percentage. The outer diameter of these tubes is in the range of 10–20 nm, and the inner diameter between 3–5 nm.

### 2.2. Nanofiber Formation

**Figure 1:**
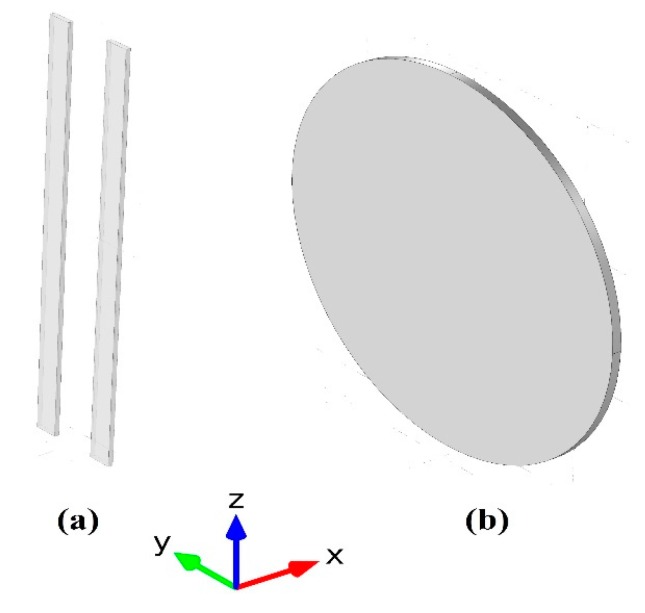
Used collector designs (**a**) 2 bars, and (**b**) traditional straight metal sheet.

Electrospinning was performed by adding 20 mL of dimethyl formamide (DMF) (Fine-Chem Limited, industrial Estate, Mumbai, India) solvent into a 3 gm polyvinylidene difluoride (PVDF) to get 15 wt % PVDF concentration. A plastic syringe tipped with a stainless steel needle was filled with 3 mL of the PVDF solution. The positive voltages came from a high voltage supply CZE1000R (Spellman, Hauppauge, NY, USA) to the metal needle, for application of bias values around (25 kV) with constant rate of (1.5 mL/h) using a syringe pump NE1000 (New Era Pump Systems, Suffolk County, NY, USA) with needle-to-collector distance of 10 cm. Random PVDF nanofibers were obtained using a normal metal plate collector covered with aluminum foil which was connected to ground. For comparison, Aligned PVDF fiber was fabricated by 2-metal bars of 1.5 cm length, as shown in Figure 1. The addition of MWCNTs with different concentration (0.1 and 0.3 wt %) was introduced by dispersing the CNTs into PVDF solution with the aid of tip sonicator (Fisher Scientific, Hampton, NH, USA) for 15 min in an ice bath to diminish the effect of heat on the CNT structure.

### 2.3. Electric Field and Orientation Analysis

Nanofibers are formed between the 2-bars due to the high electric field. We introduced a finite element simulation for the proposed collectors during the study, showing both electric and electric field vectors between the 2 bars. The finite element package COMSOL Multiphysics® Version 5 (COMSOL Inc, Stockholm, Sweden) was used to demonstrate the fields profiles. The simulations were investigated using 3D module. SEM images were analyzed using Fourier Transform through the usage of MATLAB algorithm, Image-Based Fiber Orientation Calculator by university of Minnesota-Academic Use License was obtained [32], and images were cropped to have square dimensions in pixels with elimination to scale bars to have more accurate results. The MATLAB code processes the image to generate the orientation matrix [33] defined as:

(1) $$\;\Omega =\frac{1}{Itot\;}\sum Ii\left|\begin{array}{cc} cos^{2\;}\theta i & \sin\theta i\;cos\theta i\\ \;sin\theta i\;cos\theta i & sin^{2}\theta i \end{array}\right|$$

where *Ii* is the length of the fiber, *Itot* is the total sum of fiber lengths; *θi* is the angle between each fiber and the x-axis.

The alignment scheme is determined through a factor called anisotropy index (*α*), where *α* = 0 for complete randomness of the nanofibers mats and *α* = 1 for completely aligned nanofibers. Anisotropy index is calculated from an equation relates the eigenvalues (*λ*1, *λ*2 *and λ*1 *≤ λ*2), of the orientation matrix defined as follows:

*α* = 1 − *λ*1/*λ*2 (2)

### 2.4. Characterizations Procedures

The morphology of PVDF NFs was observed by a Scanning Electron Microscope (JEOL JSM-6010LV-SEM). The diameter of NFs was analyzed using (Image J) software. The crystal phase of NFs was measured with X-ray Diffractometer (XRD) (Shimadzu Xlab 6100, Kyoto, Japan), and Fourier Transform Infra Red Spectrometer (FT-IR) (Vertex 70 FT-IR, Bruker, Billerica, MA, USA) was used for β phase content calculation. Simulation for alignment setup with orientation of electric field directions for plate and bars collectors was introduced using COMSOL finite-eliminate software. Degree of NFs alignment and anisotropy was observed using image-based fiber orientation and alignment calculator software program which is based on Fourier transform methods. Piezoelectric properties of the nanofiber mats are measured through a simple setup. The nanofiber mats of dimensions 2 cm × 2 cm were placed between two foil sheets, and pressed by different applied weights. Then, the generated voltage was detected through two connected shielded wires, pasted on the foil sheets, to a high impedance oscilloscope; Tektronix 3012 (Beaverton, OR, USA).

## 3. Results and Discussions

### 3.1. Fields Distribution Analysis

**Figure 2:**
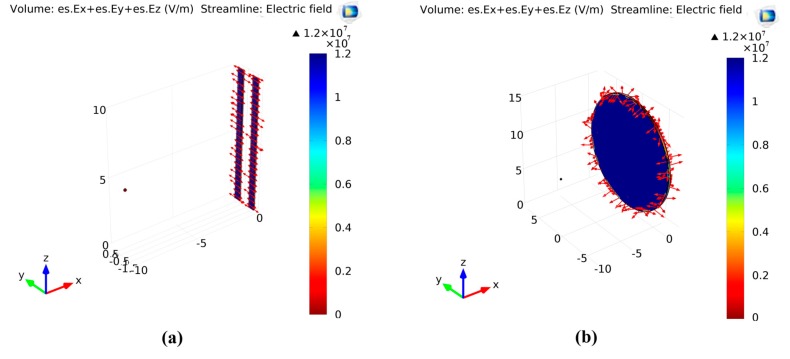
Electric field distribution for (**a**) 2 bars collector and (**b**) Conventional collector.

To show the electric field profiles, COMSOL Multiphysics was used to analyze the two collectors, to show the field profile on the normal collector and the 2 bars collector. Figure 2 shows the electric field distributions for the two types of collectors.

**Figure 3:**
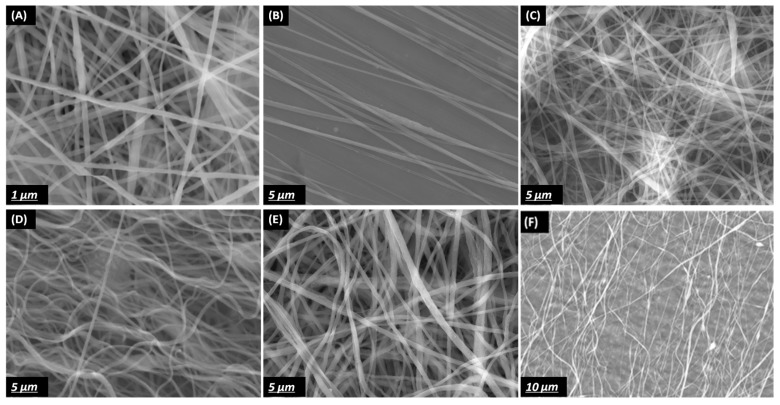
(**A**) SEM images of non-aligned PVDF 0 wt %, (**B**) CNT two-bar aligned PVDF 0 wt % CNT, (**C**) non-aligned PVDF 0.1 wt %, (**D**) CNT two-bar aligned PVDF 0.1 wt %, (**E**) CNT non-aligned PVDF 0.3 wt %CNT and (**F**) two-bar aligned PVDF 0.3 wt % CNT.

It can be observed that, in the conventional collector case, the electric field profile and vectors are not oriented in a certain direction; however, in the two-bar collector, the electric field vectors are forming stretching forces in the gap between the two-bar. This can explain the differences in the fibers’ common oriented direction. Surface morphology is shown in Figure 3.

### 3.2. Orientation Analysis 

**Figure 4:**
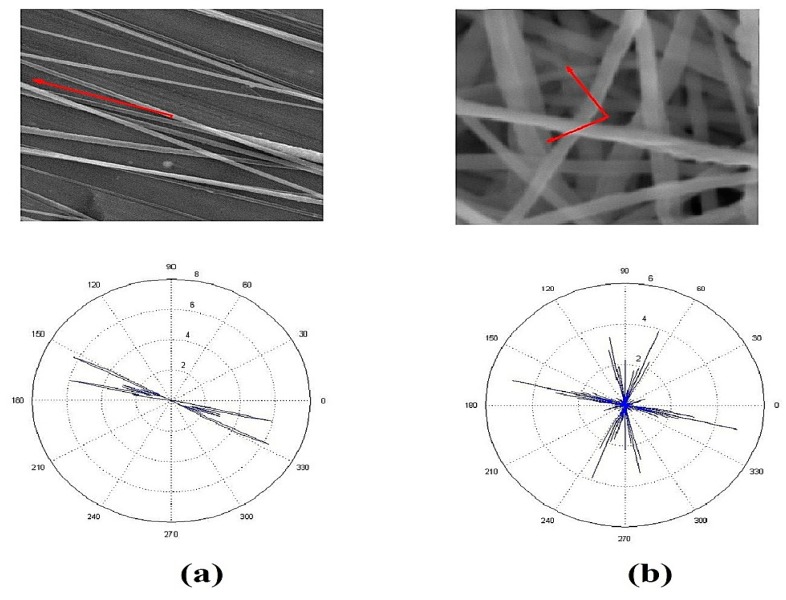
Orientation analysis for nanofibers collected on (**a**) two-bar nanofiber mat, and (**b**) conventional nanofiber mat.

Degree of alignment for the nanofibers mats (anisotropy index calculations) to prove the two-bar collector effect on the PVDF nanofibers alignment. The orientation matrix for the two-bar collector and conventional collector was obtained. Figure 4 shows the Orientation analysis for PVDF nanofibers collected on the 2 bars collector and the conventional collector. From results in Table 1, we notice an enhancement in the anisotropy index, i.e., an 82.45% improvement compared to the conventional collector.

**Figure 5:**
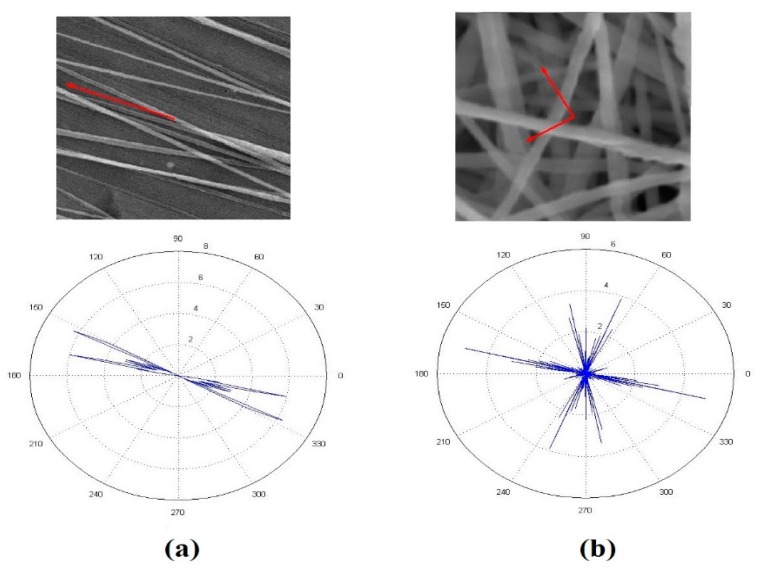
Orientation analysis for nanofibers collected on conventional collector (**a**) PVDF/0.1%CNTs nanofiber mat, and (**b**) PVDF/0.3% CNTs nanofiber mat.

**Figure 6:**
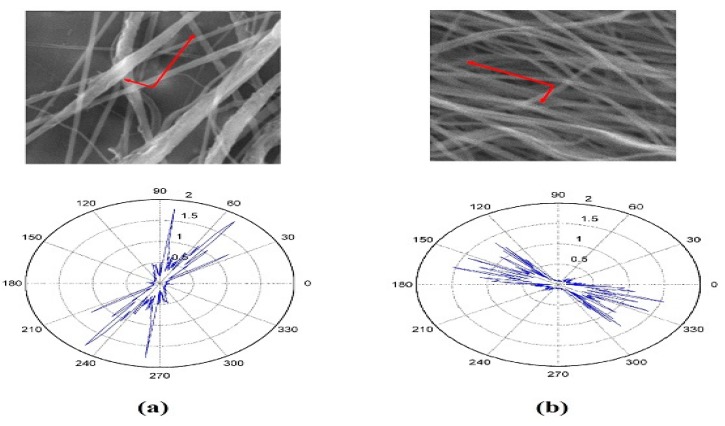
Orientation analysis for nanofibers collected on the 2 bars collector (**a**) PVDF/0.1%CNTs nanofiber mat, and (**b**) PVDF/0.3%CNTs nanofiber mat.

**Figure 7:**
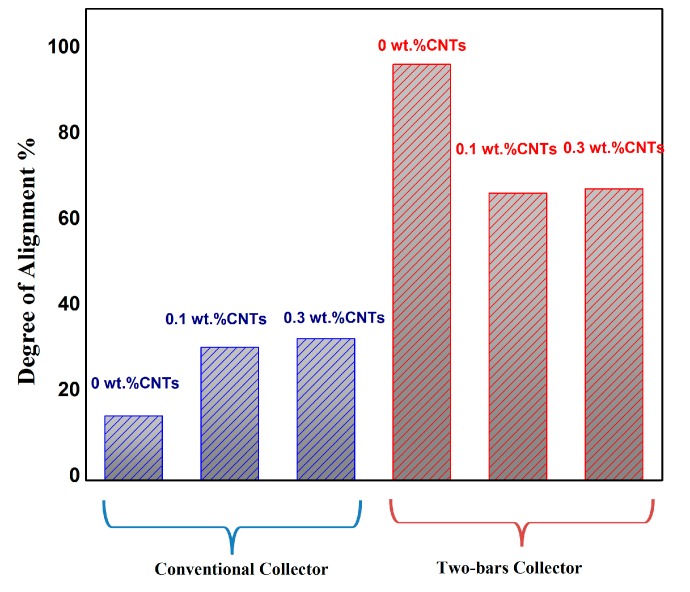
Alignment degree for nanofibers collected on the two-bars and conventional collector.

Degree of alignment for the PVDF/Carbon nanotubes (CNTs) is calculated for two samples with 0.1% and 0.3% CNTs. The anisotropy index still shows an improvement in the two-bars setup for PVDF/CNTs samples when compared to conventional collector; however, the alignment percentage decreased due to the presence of CNTs. This may be explained by the effect of CNTs charge on the orientation of electrospun nanofiber. Figure 5 shows the Orientation analysis for PVDF/CNTs nanofibers collected on the conventional collector, while Figure 6 shows the Orientation analysis for PVDF/CNTs nanofibers collected on the two-bar collector. Alignment parameters are shown in Table 2 and Table 3 for the conventional collector and the two-bar collector respectively. Figure 7 shows the complete comparison between the anisotropy index for all cases investigated through the study, it’s noticeable the 2 bars collector effect on the degree of alignment for different cases.

### 3.3. Physical Analysis

**Figure 8:**
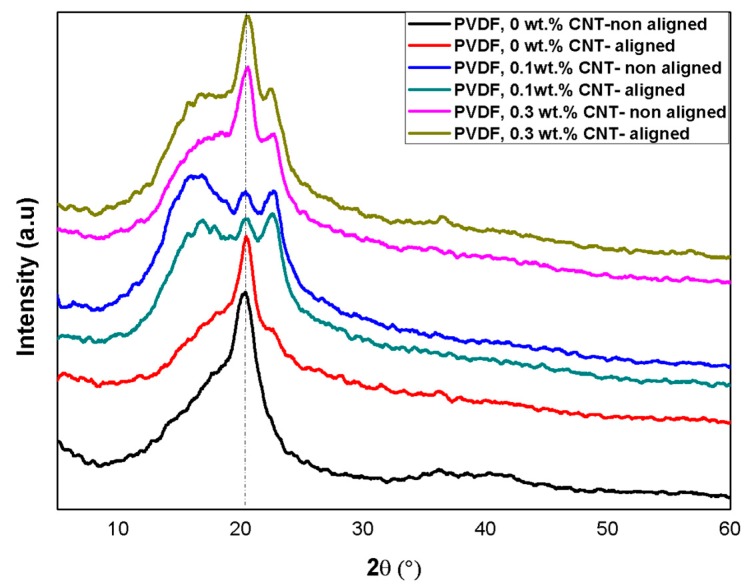
XRD analysis for aligned and non-aligned PVDF nanofiber.

XRD and FT-IR spectroscopy measurements were performed to analyze the crystal structure and content of the β-phase formation for the produced PVDF Nanofibers. The intensity of the main XRD peak corresponding to the PVDF β-phase (2*θ* = 20.6°) increased for the aligned nanofibers with two-bars as compared to random nanofiber, whereas the non-polar α-phase (2*θ* = 40°) exhibited the opposite trend (Figure 8). The observed high content of the β-phase of the aligned nanofiber can be attributed to the stretching effect due to the electric field concentration between the two bars; when nanofibers are oriented in the same direction and closely stacked, the polarization direction of the β-phase is also aligned, and thus, the β-phase content is self-reinforced (Figure 3b).

**Figure 9:**
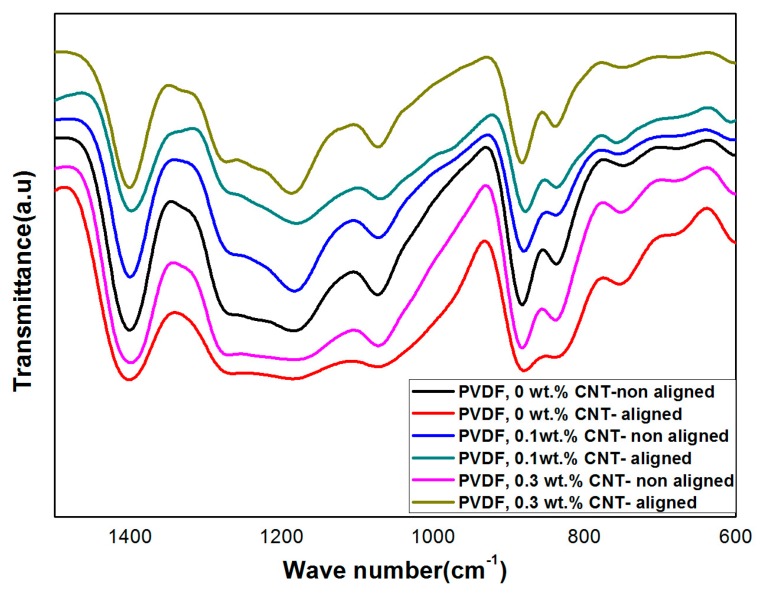
FT-IR analysis for both aligned and non-aligned PVDF nanofiber.

The IR vibrational bands observed at 612 cm^−1^ (CF_2_ bending), 766 cm^−1^ (skeletal bending), and 795 cm^−1^ (CH_2_ rocking) were due to the PVDF α-phase, while the vibrational band at 840 cm^−1^, 879 cm^−1^ and 1270 cm^−1^ corresponded to the PVDF β-phase (Figure 9). According to Beer–Lambert law for the obtained IR spectra, the PVDF β fraction can be calculated by using the following equation:

*F* (*β*) = *Aβ*/(1.3*Aα* + *Aβ*) (3)

where *F*(*β*), represents the *β* phase content, and *Aα* and *Aβ* are the absorbance at 766 and 840 cm^−1^ respectively. By calculating the previous equation according to the obtained IR result, the β-phase content for the aligned NF was 86%, whereas the random NFs exhibited β-phase contents of 74.2%.

### 3.4. Piezoelectric Characterization 

**Figure 10:**
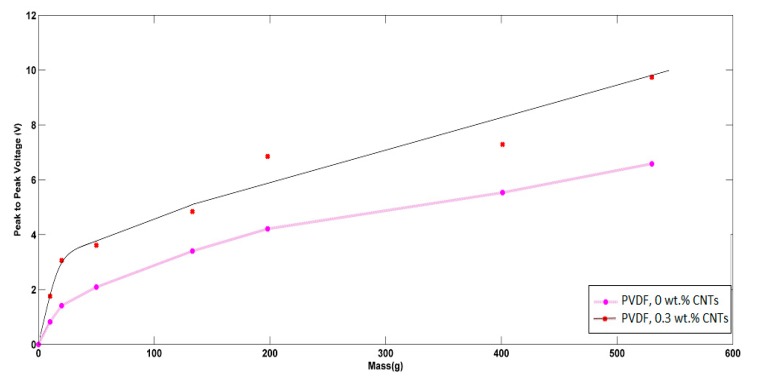
Piezoelectric response of non-aligned nanofibers at different added weight ratios of CNTs.

**Figure 11:**
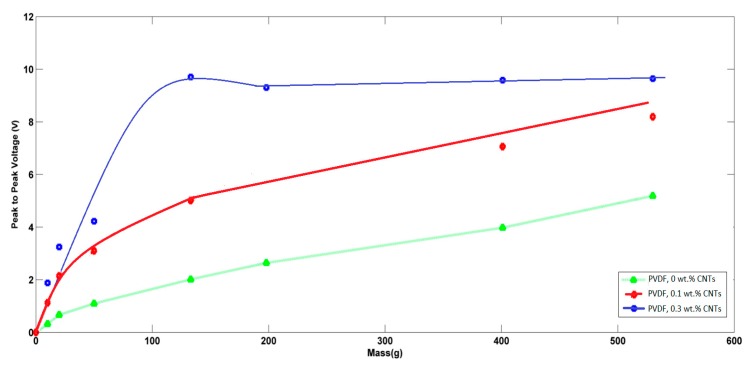
Piezoelectric response of aligned nanofibers at different added weight ratios of CNTs.

Regarding the piezoelectric analysis, each mat was exposed to pressures from different masses, and the generated peak-to-peak voltages were recorded. Both Figure 10 and Figure 11 show the piezoelectric average peak-to-peak voltage of the generated nanofibers mats in the cases of non-aligned/aligned PVDFs with added CNTs at different applied weights. In the case of non-aligned nanofibers, as expressed in Figure 10, an enhancement of piezoelectric sensitivity of PVDF nanofibers mat due to the addition of CNTs can be observed. The sensitivity of the non-aligned nanofibers mat with no added CNTs was found to be 7.2 mV/g, and increased up to 8.8 mV/g at added 0.3 wt % of CNTs. The behavior of voltage-weight relation is mostly linear in the case of no CNTs, but starts to have some non-linearity in the case of 0.3 wt % CNTs at relatively higher applied masses. In the case of aligned nanofibers, as shown in Figure 11, the alignment shows an improvement in the sensitivity of piezoelectric response, compared to the same non-aligned fibers. In the case of increasing CNT concentrations, the linearity region becomes smaller at applied masses with higher sensitivity; up to 73.8 mV/g in case of 0.3 wt % of added CNTs, but at a lower range of applied weights down, i.e., to 100 g. However, the piezoelectric response starts to be saturated at higher range of applied masses. This can be helpful in using the nanofiber mat as an electronic switch when applying higher values of masses i.e., above 150 g. To check the impact of alignment only with no added CNTs, as shown in the no added CNTs curves in both Figure 10 and Figure 11, it can be noted that the alignment leads to a better linear piezoresponse behavior due to the expected better alignment of polarized dipoles inside PVDF.

## 4. Conclusions

The work demonstrates the ability to generate highly-aligned PVDF nanofibers through static-alignment of the collector side of electrospinning setup. The synthesized PVDF nanofibers are studied with and without added in-situ CNTs. The two-bar collector system shows a great enhancement in the alignment degree for the PVDF nanofibers. Our results show that both alignment and added CNTs increased the formation of beta sheets inside the nanofibers, which correlates to a better polarizability inside the material. Consequently, the piezoelectric sensitivity of alignment PVDF doped with 0.3 wt % of CNTs was improved up to the range of 73.8 mV/g when applying weights up to 100 g, where the generated nanofibers mat can be used as a piezoelectric sensor. At higher applied weights, our synthesized nanofibers show saturation behavior which leads to the possibility of using our nanofibers as an electronic sensor with a steady output of generated voltage.

## Figures and Tables

**Table 1:** Orientation matrices (*Ω*), Eigen values (*λ*1, *λ*2) and anisotropy index (*α*) for the 2 bars collector, compared to conventional collector.

Collector	Two-bar Collector		Conventional Collector	
*Ω*	0.8627	−0.3069	0.4797	−0.0351
	−0.3069	0.1373	−0.0351	0.5203
*λ*1*,λ*2	0.0249, 0.9751		0.4595, 0.5405	
*α*	0.9745		0.1500	

**Table 2:** Orientation matrices (*Ω*)*,* Eigen values (*λ*1*, λ*2) and anisotropy index (*α*) for the conventional collector.

Collector	PVDF/0.1%CNTs		PVDF/0.3%CNTs	
*Ω*	0.4131	−0.0369	0.3983	−0.0070
	−0.0369	0.5869	−0.0070	0.6017
*λ*1*,λ*2	0.4056, 0.5944		0.3981, 0.6019	
*A*	0.3178		0.3386	

**Table 3:** Orientation matrices (*Ω*), Eigen values (*λ*1*, λ*2) and anisotropy index (*α*) for the 2 bars collector.

Collector	PVDF/0.1%CNTs		PVDF/0.3%CNTs	
*Ω*	0.3375	0.1946	0.6719	−0.1969
	0.1946	0.6625	−0.1969	0.3281
*λ*1*,λ*2	0.2465, 0.7535		0.2386, 0.7614	
*A*	0.6729		0.6866	
